# Role of G-protein-coupled receptor kinase 4 on the dysfunction of renal Mas receptor in hypertension

**DOI:** 10.1371/journal.pone.0329547

**Published:** 2025-08-05

**Authors:** Lin Chen, Jiayao Chen, Jindong Wan, Muqing Shao, Caiyu Chen, Shuo Zheng, Fuwei Zhang, Jian Yang

**Affiliations:** 1 Research Center for Metabolic and Cardiovascular Diseases, The Third Affiliated Hospital of Chongqing Medical University, Chongqing, China; 2 Department of Clinical Nutrition, The Third Affiliated Hospital of Chongqing Medical University, Chongqing, China; 3 Department of Cardiology, Daping Hospital, The Third Military Medical University, Chongqing, China; 4 Chongqing Key Laboratory for Hypertension Research, Chongqing Cardiovascular Clinical Research Center, Chongqing Institute of Cardiology, Chongqing, China; Universidade Federal do Rio de Janeiro, BRAZIL

## Abstract

The angiotensin converting enzyme 2/angiotensin-(1–7)/Mas receptor axis plays an important role in the regulation of blood pressure. G protein-coupled receptor kinase 4 (GRK4) has attracted more attentions by modulating G protein-coupled receptors and blood pressure. However, it remains unknown whether renal Mas receptor is regulated by GRK4 and its role in the pathogenesis of hypertension. Compared with Wistar-Kyoto (WKY) rats, spontaneously hypertensive rats (SHRs) exhibited impaired Mas receptor-mediated diuresis and natriuresis, which was accompanied with increased phosphorylation levels of Mas receptors. Similarly, the phosphorylation of renal Mas receptor was increased and its-induced renal effects were decreased in human (h) *GRK4γ 142V* transgenic mice relative to wild-type littermates. There was a colocalization and a direct interaction of renal Mas receptor and GRK4, which were increased in SHRs and confirmed by rigid protein–protein docking. *In vitro* studies found that treatment with the Mas receptor agonist AVE0991 inhibited Na^+^-K^+^-ATPase activity in WKY renal proximal tubule (RPT) cells, which was failed in SHR cells. GRK4 silencing decreased the phosphorylation of Mas receptor and improved the impaired Mas receptor-mediated inhibition of Na^+^-K^+^-ATPase activity in SHR RPT cells. Further study showed that ultrasound-targeted microbubble destruction-targeted renal GRK4 depletion decreased Mas receptor phosphorylation and improved its-induced diuresis and natriuresis in SHRs. These suggest that GRK4 contributes to increased renal Mas receptor phosphorylation and dysfunction in hypertension, indicating that targeting GRK4 may be a viable therapeutic approach for hypertension.

## Introduction

Hypertension is a leading risk factor for cardiovascular diseases and all-cause mortality, causing nearly 10.8 million deaths per year worldwide [1,2]. However, the pathogenesis of hypertension remains still unclear. The disturbance of sodium metabolism is a significant contributing factor in the development of hypertension. The kidney plays a crucial role in the long-term regulation of blood pressure through the maintenance of sodium homeostasis [3]. The increased sodium retention in hypertension may be attributed, at least in part, to enhanced activity of renal ion transports. This process is regulated by hormonal pathways and other systems, particularly the renin-angiotensin system (RAS) [4]. The RAS is known as a vital coordinator modulating blood pressure as well as electrolyte and fluid homeostasis. Angiotensin II (Ang II) is regarded as the main mediator of classic RAS and exerts its effects via two major receptor subtypes, namely angiotensin type 1 receptor (AT_1_R) and angiotensin type 2 receptor (AT_2_R) [5].

In recent years, the angiotensin converting enzyme 2 (ACE2)/angiotensin (Ang)-(1–7)/Mas receptor axis, a branch of the RAS, has attracted substantial attention from researchers [6,7]. Studies have shown that Ang-(1–7) and its analogues, via activating Mas receptor, exert multiple protective effects such as decreasing high blood pressure [8,9]. Especially, activation of Mas receptor exhibits beneficial effects in the kidney, including inducing natriuresis and diuresis, improving hypertensive kidney disease, protecting against renal ischemia-reperfusion injury [10,11]. Mice deficient in either ACE2 or Mas receptor exhibit more severe hypertension and renal functional impairment. These conditions were further exacerbated in mice with a dual deletion of ACE2 and Mas receptor [12]. However, the regulation of renal Mas receptor *per se* in hypertension still remains unclear.

Similar to many hormone receptors such as dopamine receptors and angiotensin receptors, the Mas receptor belongs to the G protein-coupled receptors (GPCRs) family, which is regulated by G protein-coupled receptor kinases (GRKs) [13]. GRKs, comprising seven serine/threonine protein kinases (GRK1–7), are characterized by their ability to mediate the phosphorylation and desensitization of agonist-occupied GPCRs. Numbers of studies have demonstrated the role of GRKs and their underlying mechanisms in the pathogenesis of hypertension [14,15]. Among the seven subtypes of GRKs, GRK4 has attracted more attentions in the regulation of blood pressure. Epidemiological studies show that GRK4 gene variants are correlated with reduced natriuresis and increased blood pressure, and predict the blood pressure response to antihypertensive medicines [16,17]. Moreover, our and other animal studies have demonstrated that GRK4 regulates the expression and/or phosphorylation of renal GPCRs such as dopamine receptors and Ang II receptors (AT_1_R and AT_2_R), modulates receptors-mediated renal functions [18,19]. However, it remains unclear whether renal Mas receptor, another important component of RAS, is also regulated by GRK4 and its role in the pathogenesis of hypertension. In this current study, we investigated the potential role of GRK4 in the modulation of Mas receptor and demonstrated that aberrant regulation of GRK4 on Mas receptor may be involved in the pathogenesis of hypertension.

## Methods

### Animals

Wistar-Kyoto (WKY) rats and spontaneously hypertension rats (SHRs), ranged in age from 8 to 12 weeks, were obtained from Beijing Vital River Laboratory Animal Technology Co., Ltd (Beijing, China). The human (h) *GRK4γ* wild-type (WT) and *hGRK4γ* 142V transgenic mice were generated as previously described [20]. All rodents were provided with a regular diet and maintained at the animal facilities of Animal Central of Daping Hospital. The studies were in accordance with the ethical use of animal guidelines, and approved by the Laboratory Animal Welfare and Ethics Committee of the Army Medical University.

### Animal surgical procedures

The surgical procedures of rats have been thoroughly detailed in our previous studies [21]. In brief, rats were anesthetized (intraperitoneal pentobarbital, 50 mg/kg), tracheotomized (PE-240), and placed on a heated pad to maintain body temperature. During the procedure, anesthesia was maintained via a continuous infusion of pentobarbital (0.8 mg/100 g/h). Catheters (PE-50) were inserted into the external jugular and femoral veins for fluid replacement, and left carotid artery was catheterized (PE-240) for monitoring systemic arterial pressure. After an abdominal laparotomy, the right and left ureters were exposed and catheterized (PE-10) for collecting urine. The right suprarenal artery was catheterized for vehicle (saline)/drug infusion (40 μl/h). To verify the function of Mas receptor activation, the highly selective nonpeptide Mas receptor agonist AVE0991 (0.2, 0.4, and 0.6 μg/kg/min) was used for infusion [10,22]. It should be noted that Ang-(1–7) has a relatively short half-life and low bioavailability *in vivo* due to rapid enzymatic metabolism by several proteases. To avoid this disadvantage, an Ang-(1–7) non-peptide analogue AVE0991, which has a longer half-life and increased stability due to its resistance to proteolytic enzymes, was used in this present study [23,24]. The procedures sustained approximately 60 minutes. Fluid losses incurred during the surgery were compensated with a solution of 5% albumin in normal saline at a volume equivalent to 1% body weight over 30 minutes. Following an equilibration period lasting 120 minutes, urine samples were collected for clearance measurements every 40 minutes for 5 times.

In addition, the experimental procedures involving mice have been previously detailed [20,25]. Briefly, following anesthesia (intraperitoneal pentobarbital, 50 mg/kg), the mice were catheterized (PE-10) for the purpose of monitoring blood pressure via the left carotid artery and administering fluids via the left jugular vein. Urine was collected through a suprapubic cystostomy. Vehicle/drug was infused intravenously. The procedure of urine collection and the principle of rehydration were similar with those in rats. To mitigate animal suffering, all surgical procedures were conducted under pentobarbital anesthesia. At the conclusion of the experimental procedures, mice were euthanized using pentobarbital, followed by cervical dislocation. All efforts were made to minimize animal suffering and the number of animals used.

### Cell culture

Immortalized renal proximal tubule (RPT) cells from 4–8 week old WKY rats and SHRs, obtained from Dr Ulrich Hopfer (Case Western Reserve School of Medicine, Cleveland, Ohio, U.S.A.), were cultured at 37°C in 95% of air and 5% of CO_2_ atmosphere in Dulbecco’s Modified Eagle Medium/F-12 culture media. Cells at passages 10–20 were used for experiments, as previously described [26,27]. RPT cells at 80% confluency were homogenized in ice-cold lysis buffer (PBS with 1% NP40, 0.5% sodium deoxycholate, 0.1% SDS, 1 mM EDTA,1 mM EGTA, 1 mM PMSF, 10 μg/ml aprotinin, and 10 μg/ml leupeptin), subjected to sonication, and maintained on ice for 1 h. The samples were centrifuged at 16,000g for 30 min, and the supernatants were then stored at −80°C until further use.

### GRK4-specific siRNA transfection

Small interfering RNA targeting GRK4 mRNA (GRK4 siRNA) and its control, scrambled RNA, were used as previously reported [19]. Briefly, RPT cells from SHRs were seeded in 6-well plates and cultured until reaching 60% confluence. According to the manufacturer’s instructions, 50 nmol/L GRK4-specific siRNA (RiboBio, Guangzhou, China) was mixed with 6 μL of Lipofectamine 2000 Reagent (Invitrogen Life Technologies, California, U.S.A). The nucleotide sequence of GRK4 siRNA is 5’-CCUGUAUUCUUAGACCAAAdTdT-3’ and 3’-dTdTGGACAUAAGAAUCUGGUUU-5’, and non-silencing scrambled siRNA was used as a negative control. The mixture was incubated with cells for 24 hours, after which the medium was replaced with growth medium and incubated for another 24 hours. Then, cells were harvested to assess the efficiency of siRNA treatment or used for additional experiments.

### Real-time quantitative PCR

Total RNA was isolated using RNAiso Plus (TAKARA, Tokyo, Japan) according to the manufacturer’s instructions. Using reverse transcription reagent (Bio-Rad Laboratories, Hercules, CA, U.S.A.), a total of 2 µg of total RNA was used as a template to synthesize cDNA, which served for the amplification of target genes. The specific primers for gene expression analysis are listed in S1 Table. The PCR was performed under the following conditions: 95 °C for 3 minutes; 40 cycles of 95 °C for 10 seconds and 62 °C for 30 seconds; and 62 °C for 10 seconds. Gene expression levels were normalized to GAPDH expression.

### Immunoblotting

The protein concentration of cell lysates or kidney homogenates was determined by Bicinchoninic Acid Assay (BCA) protein concentration test kit (Beyotime, shanghai, China). Samples containing 50 μg of total protein were separated by 10% sodium dodecyl sulfate polyacrylamide gel electrophoresis (SDS‒PAGE), transferred onto nitrocellulose membranes, and then blocked with protein free rapid blocking buffer (Epizyme PS108, Shanghai, China) at room temperature for 10 min. After washing, the blotted membranes were probed with the primary antibodies overnight at 4 °C and then incubated with infrared-labeled secondary antibodies at room temperature for 1 h. The antibodies used in the present study are detailed in S2 Table. The protein complex bands were visualized using the Odyssey Infrared Imaging System (Li-Cor Biosciences, Lincoln, NE). The protein expression was normalized to GAPDH or β-tubulin expression. The lack of GAPDH use in some figures was due to very small difference between 37 and 42 kDa.

### Sample staining and STED

The kidney samples from WKY rats and SHRs were sectioned into 4 μm thick slices. The slices were then incubated overnight at 4 °C with primary antibodies including rabbit anti-Mas receptor antibody and mouse anti-GRK4 antibody. Subsequently, samples were incubated with Abberior STAR ORANGE goat anti-rabbit IgG and Abberior STAR RED goat anti-mouse IgG, which were used specifically for stimulated emission depletion (STED) microscope imaging. The antibodies used in the present study are detailed in S2 Table. In addition, the nuclei in the samples were stained with 4’, 6-diamidino-2-phenylindole (DAPI). Samples were imaged with a STEDYCON (Abberior, Göttingen, Germany) on an inverted microscope (Olympus, Shinjuku, Tokyo) [28].

### Immunoprecipitation

Immunoprecipitation was performed as described in our previous studies [29]. In brief, equal amounts of lysate (300 μg protein/mL supernatant) were incubated with an anti-GRK4 antibody (5 μg/mL) or an anti-phosphoserine antibody (5 μg/mL) for 1 h and with protein-A/G plus-agarose at 4°C for overnight. The immunoprecipitates were then subjected to immunoblotting with an anti-Mas receptor antibody. In addition, IgG (negative control) and the anti-Mas receptor antibody (positive control) were also used to determine the specificity of the bands on the immunoblots.

### Molecular docking analysis

To further explore the possible interaction of the Mas receptor and GRK4, rigid protein–protein docking was conducted by using GRAMM-X (http://gramm.compbio.ku.edu/). The protein structural domains of Mas receptor and GRK4 were sourced from the Protein Data Bank (PDB; http://www.rcsb.org/). For detailed analysis of protein-protein direct interactions, Pymol (version 2.4) and PDBePISA (https://www.ebi.ac.uk/pdbe/pisa/) were employed.

### Na^+^-K^+^-ATPase activity assay

The Na^+^-K^+^-ATPase activity in the crude membrane fraction of RPT cells was investigated using a commercially available Na^+^-K^+^-ATPase Assay Kit (Solarbio, Beijing, China), according to the manufacturer’s protocol. Na^+^-K^+^-ATPase activity was calculated as the difference between the total ATPase activity and the ouabain-insensitive ATPase activity. The results were collected and expressed as μmol phosphate released per mg protein per min.

### Ultrasound-targeted microbubble destruction

Microbubbles carrying GRK4 siRNA were prepared by poly-L-lysine and electrostatic adsorption, as we have reported previously [30]. Briefly, rats were anesthetized with 2% pentobarbital sodium (50 mg/kg). The microbubbles were oscillated form microbubble suspensions and administrated into the lateral tail vein under the control of syringe pump. Only the right kidney of each rat was treated with ultrasound-targeted microbubble destruction (UTMD), which was irradiated using the 9L4 linear array probe of an ultrasound imaging system (42 frames/SEC, MI = 0.9, frequency = 7.00 MHz) for 5 min. The UTMD-mediated GRK4 siRNA delivery was carried out every three days. Totally, each rat received six treatments.

### Statistical analysis

All data are expressed as mean ± SD and points represent individual animals. Statistical comparisons are performed using repeated measures ANOVA (or paired *t*-test when only two groups were compared), or one-way ANOVA with Holm-Sidak test (or *t*-test when only two groups were compared) as appropriate. The Shapiro-Wilk test was used to determine normal or non-normal distribution prior ANOVA. Values of **P* *< 0.05 were considered statistically significant.

## Results

### Increased Mas receptor phosphorylation and impaired its-mediated natriuretic and diuretic effects in renal cortex of SHRs

**Fig 1:**
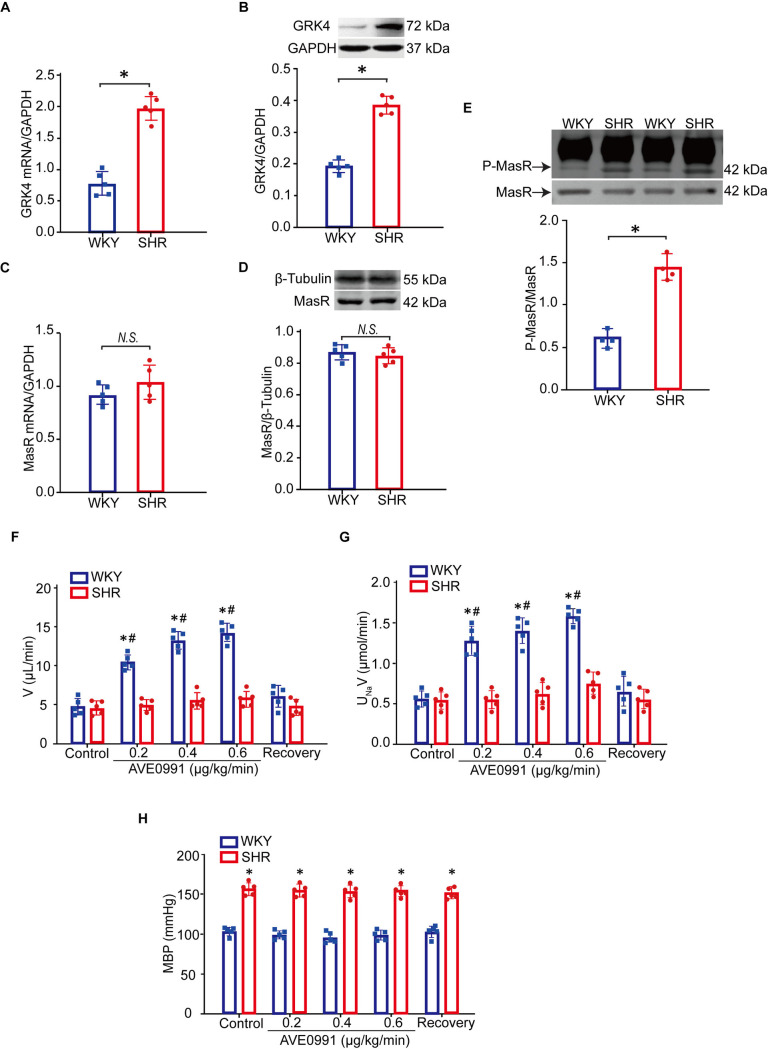
Mas Receptor Phosphorylation and its-mediated Effects in Renal Cortex of WKY Rats and SHRs. A) and B) GRK4 mRNA (A) and protein (B) levels in renal cortex of WKY rats and SHRs (*P < 0.05, compared with WKY; *n* = 5/group). C) and D) Mas receptor (MasR) mRNA (C) and protein (D) expressions in renal cortex of WKY rats and SHRs. (*N.S*. means no significance, compared with WKY; *n* = 5/group). E) Mas receptor (MasR) phosphorylation in renal cortex of WKY rats and SHRs. Mouse anti-phosphoserine antibody was used for immunoprecipitation. The immune complexes were precipitated using agarose-A/G beads and immunoblotted for Mas receptor (*P < 0.05, compared with WKY; *n* = 4/group). F) and G) Effects of the intrarenal arterial infusion of the Mas receptor non-peptide agonist AVE0991 (0.2, 0.4, and 0.6 μg/kg/min) on urinary flow (V) (F) and urinary sodium excretion (UNaV) (G) in WKY rats and SHRs (*P < 0.05, compared with WKY control; ^#^*P* < 0.05, compared with SHRs, *n* = 5/group). H) The mean arterial blood pressure (MBP) in pentobarbital-anesthetized WKY rats and SHRs during the infusion of AVE0991 (*P < 0.05, compared with WKY, *n* = 5/group).

The renal cortical levels of GRK4 and Mas receptor were determined in WKY rats and SHRs. Results showed that both mRNA and protein levels of GRK4 were elevated in the renal cortex of SHRs relative to WKY rats (Figs 1A and 1B). The renal cortex of WKY rats and SHRs exhibited comparable levels of Mas receptor mRNA and protein expression (Figs 1C and 1D). However, the phosphorylation of Mas receptor was notably increased in the renal cortex of SHRs (Fig 1E).

To assess whether increased Mas receptor phosphorylation affects its-mediated physiological renal function, AVE0991, a nonpeptide analogue of Ang-(1–7), was used [8,31]. Administration of the Mas receptor agonist AVE0991 (0.2, 0.4, and 0.6 μg/kg/min) in WKY rats led to a dose-dependent elevation in sodium excretion and urine flow (Figs 1F and 1G). However, these effects induced by AVE0991 were failed in SHRs, implying that renal Mas receptor mediated-natriuretic and -diuretic effects was impaired in SHRs. During the intrarenal infusion process, treatment with AVE0991 exhibited no impact on mean arterial blood pressure in either WKY rats or SHRs (Fig 1H), indicating that the Mas receptor-mediated renal effects were independent of blood pressure alterations.

### *hGRK4γ* 142V transgenic mice displayed impaired Mas receptor-induced renal effects

**Fig 2:**
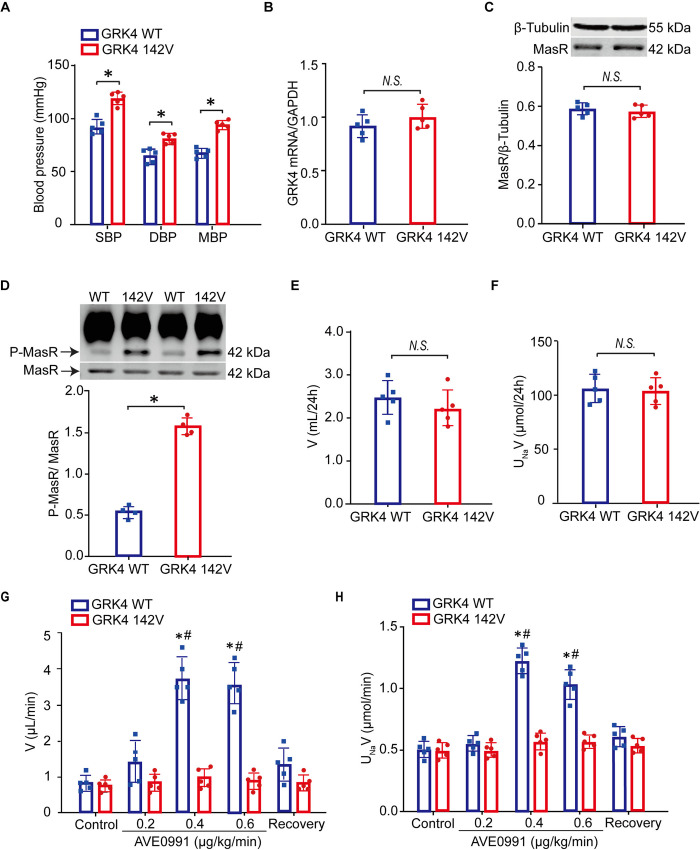
The phosphorylation of Mas receptor and its induced-effects in *hGRK4* *γ* WT and 142V transgenic mice. (A) Systolic, diastolic, and mean arterial blood pressure (SBP, DBP, and MBP) in *hGRK4γ* wild-type (WT) and *hGRK4γ* 142V transgenic mice (*P < 0.05, compared with WT, *n* = 5/group). B) and C) Mas receptor (MasR) mRNA (B) and protein (C) levels in *hGRK4γ* WT and *hGRK4γ* 142V transgenic mice (*N.S*. means no significance, compared with WT; *n* = 5/group). D) Mas receptor (MasR) phosphorylation in renal cortex of *hGRK4γ* WT and *hGRK4γ* 142V transgenic mice,and mouse anti-phosphoserine antibody was used for immunoprecipitation. The immune complexes were precipitated using agarose-A/G beads and immunoblotted for Mas receptor (*P < 0.05, compared with WT; *n* = 4/group). E) and F) 24h urine volume (V) (E) and urinary sodium excretion (UNaV) (F) in *hGRK4γ* 142V and WT transgenic mice (*N.S*. means no significance, compared with WT; *n* = 5/group). G) and H) Effects of the intravenous infusion of the Mas receptor non-peptide agonist AVE0991 (0.2, 0.4, and 0.6 μg/kg/min) on urinary flow (V) (G) and urinary sodium excretion (UNaV) (H) in *hGRK4γ* 142V and WT transgenic mice (*P < 0.05, compared with WT control; ^#^*P* < 0.05, compared with 142V, *n* = 5/group).

To further investigate whether the enhanced GRK4 activity was responsible for renal Mas receptor dysfunction, GRK4 variant *hGRK4γ* 142V transgenic mice were generated. Results showed that systolic, diastolic, and mean arterial blood pressures (SBP, DBP, and MBP) were remarkably increased in *hGRK4γ* 142V transgenic mice compared to *hGRK4γ* WT transgenic counterparts (Fig 2A). There was no difference of Mas receptor mRNA and protein expression in the renal cortex between the two group mice (Figs 2B and 2C). To our interesting, the phosphorylation of Mas receptor was significantly increased in the renal cortex of *hGRK4γ* 142V transgenic mice relative to *hGRK4γ* WT transgenic mice (Fig 2D), suggesting that the increased Mas receptor phosphorylation might be due to the enhanced GRK4 activity.

We next explored the Mas receptor-mediated natriuresis in *hGRK4γ* 142V transgenic mice. Basal 24 h sodium excretion and urine flow were similar between the two group mice (Figs 2E and 2F); however, administration of Mas receptor agonist AVE0991 (0.2, 0.4, and 0.6 μg/kg/min) elicited natriuretic and diuretic effects in *hGRK4γ* WT transgenic mice, which were absent in *hGRK4γ* 142V transgenic mice (Figs 2G and 2H). These results suggested that the elevated phosphorylation of Mas receptor might be involved in its impaired renal functions in *hGRK4γ* 142V transgenic mice.

### Renal GRK4 interacted with Mas receptor

**Fig 3:**
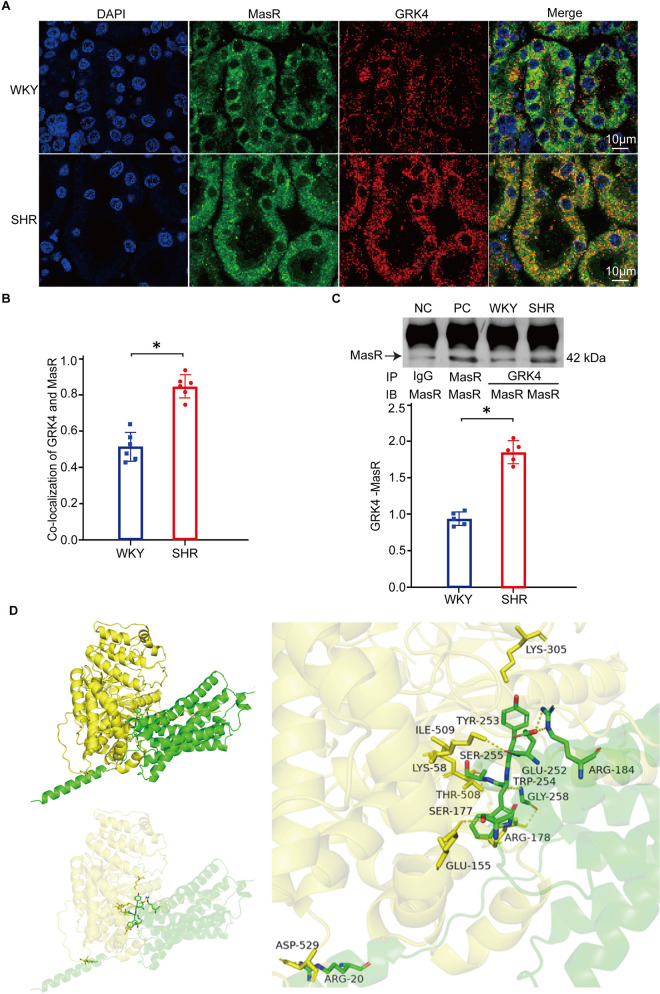
Interaction between Mas receptor and GRK4 in the renal cortex of WKY rats and SHRs. A) and B) Mas receptor and GRK4 co-localization (A) in the renal cortices of WKY rats and SHRs, which were quantified (B) by Image Pro Plus software. Colocalization appears as yellow after merging the images of Abberior STAR ORANGE-tagged MasR (Green) and Abberior STAR RED-tagged GRK4 (red) (scale bar, 10 μm. *P < 0.05, compared with WKY, *n* = 6 fields *per* group from 3 rats, 2 fields *per* rat). C) Co-immunoprecipitation of Mas receptor (MasR) and GRK4 in the renal cortices of WKY rats and SHRs (*P < 0.05, compared with WKY, *n* = 5/group). NC, negative control; PC, positive control. D) The docking model between GRK4 and Mas receptor and its molecular conformation diagram (GRK4, yellow; Mas receptor, green).

Whether Mas receptor and GRK4 in the renal cortex of WKY rats and SHRs has direct interaction was investigated. Results of STED super-resolved microscopy showed that there was colocalization of Mas receptor and GRK4 in the kidneys of WKY rats and SHRs (Figs 3A and 3B). This was further verified by coimmunoprecipitation, which found the direct interaction of renal Mas receptor and GRK4 in both WKY rats and SHRs (Fig 3C). More importantly, we also found that the colocalization and direct interaction of Mas receptor and GRK4 were obviously increased in the kidneys of SHRs compared to WKY rats (Figs 3A-3C), which could potentially be a contributing factor to the augmented phosphorylation of Mas receptor in the renal cortex of SHRs. Furthermore, the direct interaction of renal Mas receptor and GRK4 was confirmed by rigid protein–protein docking. The results showed that Mas receptor and GRK4 formed hydrogen bonds through amino acid residue sites such as ARG 20-ASP 529 and SER 177-GLU 155 (S3 Table), revealing that proteins Mas receptor and GRK4 formed a stable protein docking model (Fig 3D), which is consistent with the results of coimmunoprecipitation.

### GRK4 silencing recovered Mas receptor-mediated inhibition of Na^+^-K^+^-ATPase activity in SHR RPT cells

**Fig 4:**
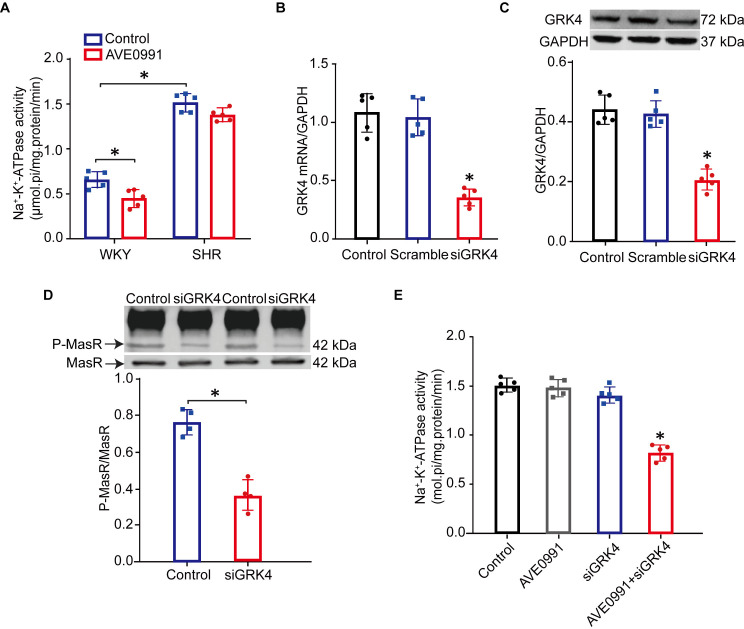
Mas receptor phosphorylation and its-mediated effect on Na⁺ -K⁺ -ATPase activity in GRK4 siRNA-treated SHR RPT Cells. A) Effect of Mas receptor non-peptide agonist AVE0991 on Na^+^-K^+^-ATPase activity in RPT cells from WKY rats and SHRs. Cells were incubated with AVE0991 (40 nM) or vehicle for 30 min (*P < 0.05, compared with WKY control; *n* = 5/group). B) and C) Effect of GRK4-specific siRNA on GRK4 mRNA (B) and protein (C) expressions in RPT cells from SHRs (*P < 0.05, compared with control; *n* = 5/group). D) The phosphorylation of Mas receptor (MasR) in SHR RPT cells transfected with GRK4 siRNA. The samples were immunoprecipitated with anti-phosphoserine antibody and then immunoblotted with anti-Mas receptor antibody (*P < 0.05, compared with control; *n* = 4/group). E) Effect of the Mas receptor non-peptide agonist AVE0991 (40 nM) on Na^+^-K^+^-ATPase activity in GRK4 siRNA-treated SHR RPT cells. Cells were incubated with AVE0991 (40 nM) or vehicle for 30 min (*P < 0.05, compared with control; *n* = 5/group).

To further determine the effect of GRK4 on the regulation of renal Mas receptor, we next examined whether specific GRK4 depletion could rescue the functional deficits of Mas receptor in SHR PRT cells. Compared to WKY RPT cells, the basal Na^+^-K^+^-ATPase activity was elevated in SHR RPT cells; treatment with AVE0991 (40 nM), a Mas receptor agonist, inhibited Na^+^-K^+^-ATPase activity in WKY RPT cells (Fig 4A). However, this was failed in SHR RPT cells, indicating that the impaired Mas receptor-mediated natriuresis in SHRs might be, at least in part, due to its diminished capacity to suppress Na^+^-K^+^-ATPase activity. More importantly, GRK4 silencing decreased the phosphorylation of Mas receptor in SHR RPT cells, which was accompanied with the reduced GRK4 expression (Figs 4B-4D). This was of physiological significance because GRK4 siRNA treatment improved the impaired AVE0991-activated Mas receptor-mediated inhibition of Na^+^-K^+^-ATPase activity to some extent in SHR RPT cells (Fig 4E). These suggested that GRK4 silencing in SHR RPT cells attenuates the increased phosphorylation of Mas receptor and recovers the impaired Mas receptor-mediated inhibition of Na^+^-K^+^-ATPase activity.

### Renal GRK4 depletion improved Mas receptor-induced natriuresis in SHRs

**Fig 5:**
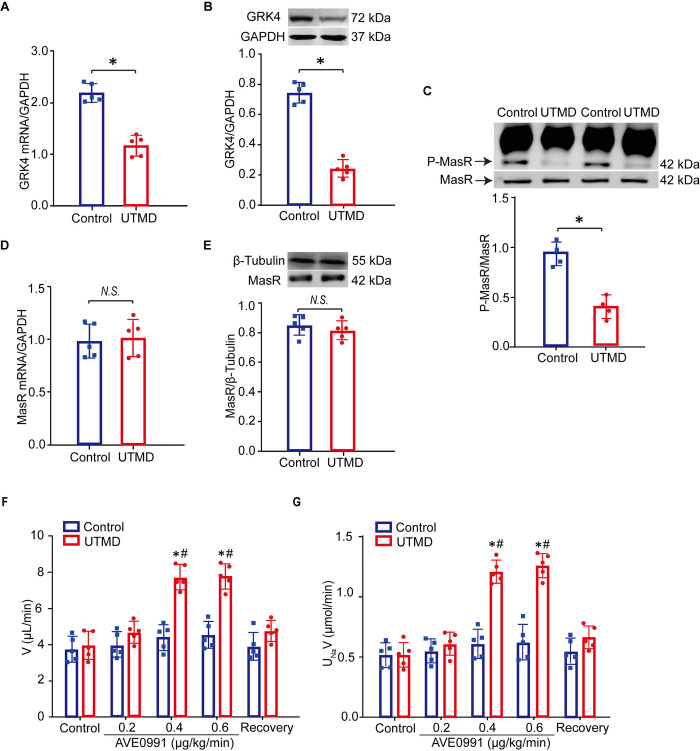
Renal GRK4 knockdown impacted Mas receptor phosphorylation and Its-induced renal function in SHRs. A) and B) Effect of ultrasound-targeted microbubble destruction (UTMD)-mediated GRK4-siRNA delivery on renal GRK4 mRNA (A) and protein (B) expressions in SHRs (*P < 0.05 compared with control, *n* = 5/group). C) Effect of UTMD-mediated GRK4-siRNA delivery on renal Mas receptor (MasR) phosphorylation in SHRs. The samples were immunoprecipitated with anti-phosphoserine antibody and then immunoblotted with anti-Mas receptor antibody (*P < 0.05, compared with control; *n* = 4/group). D) and E) Effect of renal GRK4 knockdown on renal Mas receptor (MasR) mRNA (D) and protein (E) levels in SHRs (*N.S*. means no significance, compared with control; *n* = 5/group). F) and G) Effect of the intrarenal arterial infusion of the Mas receptor non-peptide agonist AVE0991 (0.2, 0.4, and 0.6 μg/kg/min) on urinary flow (V) (F) and urinary sodium excretion (UNaV) (G) in SHRs treated with renal GRK4 knockdown (*P < 0.05, compared with control; ^#^*P* < 0.05, compared with control + UTMD, *n* = 5/group).

The effect of specific silencing of GRK4 on the regulation of Mas receptor function in RPT cells was further validated in *in vivo* studies. SHRs were administrated with UTMD for 4 weeks, by which GRK4 siRNA was delivered into the kidney. Results showed that both renal GRK4 mRNA and protein levels were significantly decreased following UTMD-targeted renal GRK4 silencing (Figs 5A and 5B), which was accompanied with reduced phosphorylation levels of Mas receptor (Fig 5C). However, renal GRK4 silencing did not alter the mRNA and protein levels of Mas receptor in the renal cortex of SHRs (Figs 5D and 5E). The decreased Mas receptor phosphorylation was of functional significance. UTMD-delivered renal GRK4 siRNA ameliorated the impaired Mas receptor-induced natriuresis and dieresis (Figs 5F and 5G). These suggested that UTMD-targeted renal GRK4 depletion decreases Mas receptor phosphorylation and improves Mas receptor-induced natriuresis in SHRs.

## Discussion

The RAS plays a pivotal role in the pathophysiology of hypertension [32]. As the momentous component of the RAS, the ACE2-Ang-(1–7)-Mas axis has been shown to play an important role in regulating blood pressure and emerged as a promising target for treatment of hypertension [33,34]. As the effector of Ang-(1–7), Mas receptor mediates the effects of this axis, which counteracts most deleterious actions of the RAS in the blood vessel and kidney [35]. Activation of Mas receptor ameliorates nitric oxide bioavailability and microvascular remodeling during chronic AT_1_R blockade [36], improves renal hemodynamics [37], protects against renal ischemia-reperfusion injury [38], and induces natriuresis and diuresis [39]. In the current study, our results showed that SHRs had higher phosphorylated levels, but not mRNA and protein expressions, of Mas receptor in the renal cortex than WKY rats. Further studies demonstrated that the Mas receptor agonist AVE0991-mediated natriuresis and diuresis effects were impaired in SHRs relative to normotensive WKY rats. These indicated that the weakened Mas receptor-mediated renal function may be, at least in part, attributed to its increased phosphorylation.

Numbers of studies have shown that GRK4 plays an important role in regulating renal GPCRs (e.g., dopamine receptor), consequently modulating sodium balance and blood pressure [40]. Genetic studies in humans also display that GRK4 is correlated with hypertension and blood pressure response to antihypertensive medicines [16,17]. Especially, our previous studies have reported that constitutively active GRK4 variants (e.g., A142V) leads to the dysfunction of renal AT_1_R and AT_2_R [15,41], the two main effectors of Ang II, which is involved in the pathogenesis of hypertension. However, it is still unknown whether renal Mas receptor is also regulated by GRK4 and its role in the pathogenesis of hypertension. Studies have reported that Mas receptor interacts with AT_1_R [42], AT_2_R and dopamine D_1_ receptor in the kidney [39,43], which contributes to the maintenance of sodium homeostasis. Thus, we speculated that the impaired renal Mas receptor function induced by its hyperphosphorylation may be due to increased GRK4 expression and/or activity. Our current study found that compared with control mice, *hGRK4γ* 142V transgenic mice had increased renal Mas receptor phosphorylation. Moreover, the increased Mas receptor phosphorylation was of functional significance because Mas receptor-mediated natriuresis and diuresis were decreased in *hGRK4γ* 142V transgenic mice. In addition, UTMD-delivered renal GRK4 siRNA reduced the levels of Mas receptor phosphorylation and increased the attenuated Mas receptor-induced renal functions in SHRs.

It should be noted that GRK4 exerts different effects in renal RAS receptors. *hGRK4γ* 142V increase the expression and activity of renal AT_1_R via renal histone deacetylase type 1 inhibition, which causes greater pressor response to angiotensin II [18]. However, GRK4 reduces renal AT_2_R-mediated diuresis and natriuresis via increasing AT_2_R phosphorylation, not by decreasing its expression [19]. Our results showed that GRK4 attenuated the Mas receptor-induced natriuresis and diuresis by aggravating its phosphorylation, which is similar to the effect of GRK4 in the regulation of AT_2_R, not AT_1_R. In addition, we also reported that increased GRK4 activity causes the elevated D_1_R phosphorylation and impairs its renal function [44]. These suggest that the regulation of GRK4 on renal GPCRs may be due to different mechanisms, at least including modulation of receptor expression and/or phosphorylation.

In addition, in the present study, we found the colocalization and direct interaction of Mas receptor and GRK4 in the kidneys of WKY rats and SHRs via STEDYCON and coimmunoprecipitation, which were increased in SHRs. Moreover, we also detected the direct interaction of Mas receptor and GRK4 by molecular docking analysis. These results demonstrated that the augmented interaction of renal Mas receptor and GRK4 may lead to the increased phosphorylation of Mas receptor and subsequently its dysfunction in the kidney of SHRs.

There are several limitations in our current study. First, GRK4 global transgenic 142V mice were used in this study. It would be better to using kidney-specific transgenic mice to demonstrate its renal effects. Second, we only predicted potential binding sites of renal Mas receptor and GRK4 by rigid protein–protein docking, which was not further confirmed. Third, it is not clear that the regulatory effect of renal GRK4 on Mas receptor exists in other important organs and tissues, such as blood vessels and heart, involved in blood pressure regulation.

**Fig 6:**
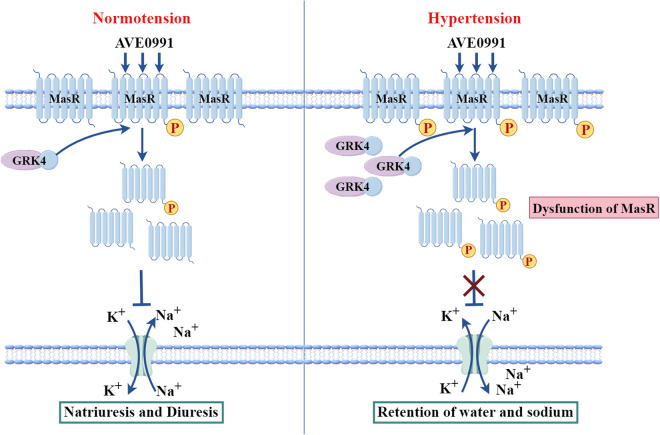
Schematic diagram of GRK4-mediated regulation of Mas receptor in the kidney under hypertensive state. In the physiological state, GRK4 modulates renal cortical Mas receptor phosphorylation at normal levels. Stimulation with the Mas receptor non-peptide agonist AVE0991 inhibits Na^+^-K^+^-ATPase activity, thereby promoting natriuretic and diuretic function. In hypertensive state, the increased expression of GRK4 in the kidney causes hyperphosphorylation of Mas receptor, which leads to impaired its-mediated inhibition of Na^+^-K^+^-ATPase activity, and ultimately causes sodium and water retention.

In conclusion, our current study found that GRK4 impairs renal Mas receptor-mediated natriuresis and diuresis by elevating its phosphorylation (Fig 6). Renal GRK4 depletion recovers the impaired renal functions of Mas receptor in SHRs. These findings indicate that targeting GRK4 may provide a novel therapeutic approach to improve the impaired Mas receptor-mediated renal functions.

## Supporting information

S1 Table Specific primers designed for the analysis of gene expression in rats and mice utilized in RT-qPCR. (DOCX)

S2 Table Details of antibodies used for immunoblotting and Immunofluorescence staining. (DOCX)

S3 Table Analysis of the interaction between GRK4 and Mas receptor derived from PDBePISA (https://www.ebi.ac.uk/pdbe/pisa/). (DOCX)

S1 Raw Images Original uncropped blot images. (PDF)
