# Supplementary material for: Role of G-protein-coupled receptor kinase 4 on the dysfunction of renal Mas receptor in hypertension
Source: PLoS One. 2025 Aug 5;20(8):e0329547. doi: 10.1371/journal.pone.0329547 (PMC12324092; doi:10.1371/journal.pone.0329547)
Supplement: S1 Table — (DOCX) [file pone.0329547.s001.docx]

**Supplemental Tables**

**S1 Table.** **Specific primers designed for the analysis of gene expression in rats and mice utilized in RT-qPCR**

| Gene | Direction | Sequence | Species |
| --- | --- | --- | --- |
| GRK4 | Forward | GAAGTCAGAAGTCCCCTTACCAGAG | Mouse |
| GRK4 | Reverse | GGAGACAGAAAACCCAGCACATTC | Mouse |
| GRK4 | Forward | CCACCTTTCTGTCCTGATCCTGAG | Rat |
| GRK4 | Reverse | CCCTGTCGCAAATTGAGTGTAGAAG | Rat |
| MasR | Forward | ATCAGTGTGGAGAGGTGCCTATCG | Mouse |
| MasR | Reverse | ACGAATGCTGACTGGTGCTTGG | Mouse |
| MasR | Forward | CATCATCTTCATAGCCATCCTCAGC | Rat |
| MasR | Reverse | GTTCTTCCGTATCTTCACCACCAAG | Rat |
| GAPDH | Forward | AGGTCGGTGTGAACGGATTTG | Mouse |
| GAPDH | Reverse | TGTAGACCATGTAGTTGAGGTCA | Mouse |
| GAPDH | Forward | CAAGTTCAACGGCACAGTCAAGG | Rat |
| GAPDH | Reverse | ACATACTCAGCACCAGCATCACC | Rat |
