# Supplementary material for: Role of G-protein-coupled receptor kinase 4 on the dysfunction of renal Mas receptor in hypertension
Source: PLoS One. 2025 Aug 5;20(8):e0329547. doi: 10.1371/journal.pone.0329547 (PMC12324092; doi:10.1371/journal.pone.0329547)
Supplement: S2 Table — (DOCX) [file pone.0329547.s002.docx]

**S2 Table.** **Details of antibodies**

| Primary / Secondary antibody | | Source/ Product | Company |
| --- | --- | --- | --- |
| Anti-GRK4 | Rabbit | | ABclonal |
| Anti-MasR | Rabbit | | Proteintech |
| Anti-phosphoserine | Rabbit | | Abcam |
| Anti-GAPDH | Mouse | | Proteintech |
| Anti-β-tubulin | Mouse | | Zenbio |
| Goat anti-mouse | IRDye 680RD | | LI-COR biosciences |
| Goat anti-rabbit | IRDye 800CW | | LI-COR biosciences |
| Anti-GRK4 | Mouse | | Santa Cruz Biotechnology |
| Anti-MasR | Mouse | | Santa Cruz Biotechnology |
| Goat anti-rabbit | Abberior STAR ORANGE | | Abberior |
| Goat anti-mouse | Abberior STAR RED | | Abberior |
| Anti- NKAα1 | Rabbit | | Proteintech |
| Anti-Calnexin | Rabbit | | Proteintech |
