# Supplementary material for: Role of G-protein-coupled receptor kinase 4 on the dysfunction of renal Mas receptor in hypertension
Source: PLoS One. 2025 Aug 5;20(8):e0329547. doi: 10.1371/journal.pone.0329547 (PMC12324092; doi:10.1371/journal.pone.0329547)
Supplement: S3 Table — (DOCX) [file pone.0329547.s003.docx]

**S3 Table. Analysis of the Interaction Between GRK4 and Mas receptor derived from PDBePISA (https://www.ebi.ac.uk/pdbe/pisa/)**

**Hydrogen bonds**

| **No** | **Mas receptor** | **Dist.[A]** | **GRK4** |
| --- | --- | --- | --- |
| 1 | A:ARG  20[NH2] | 2.28 | C:ASP 529[OD2] |
| 2 | A:SER 177[OG] | 2.53 | C:GLU 155[OE1] |
| 3 | A:ARG 184[NH1] | 3.01 | C:ASN 562[OD1] |
| 4 | A:GLU 252[O] | 2.98 | C:LYS  58[NZ] |
| 5 | A:GLU 252[OE1] | 3.77 | C:LYS  58[NZ] |
| 6 | A:TYR 253[OH] | 2.74 | C:LYS 305[NZ] |
| 7 | A:TRP 254[O] | 3.84 | C:THR 508[OG1] |
| 8 | A:SER 255[O] | 3.41 | C:ILE 509[N] |
| 9 | A:GLY 258[O] | 2.43 | C:ARG 178[NH1] |
