# Supplementary figures and images for: Role of G-protein-coupled receptor kinase 4 on the dysfunction of renal Mas receptor in hypertension
Source: PLoS One. 2025 Aug 5;20(8):e0329547. doi: 10.1371/journal.pone.0329547 (PMC12324092; doi:10.1371/journal.pone.0329547)

Figure 1B

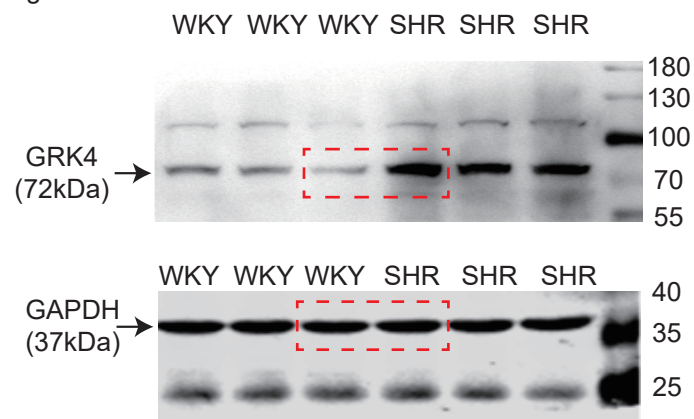

Figure 1D

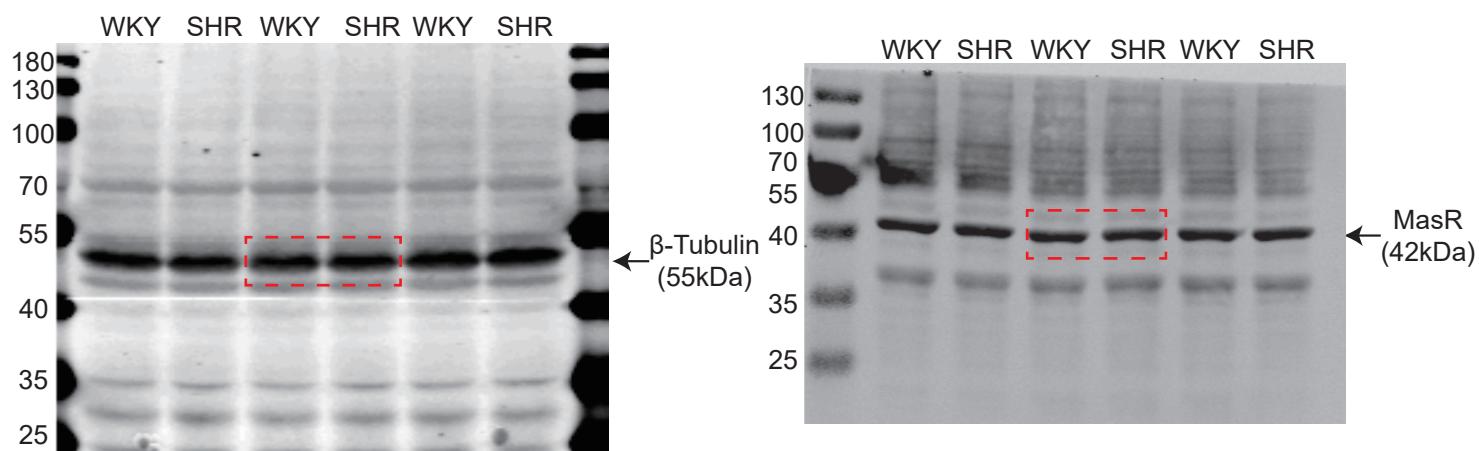

Figure 1E

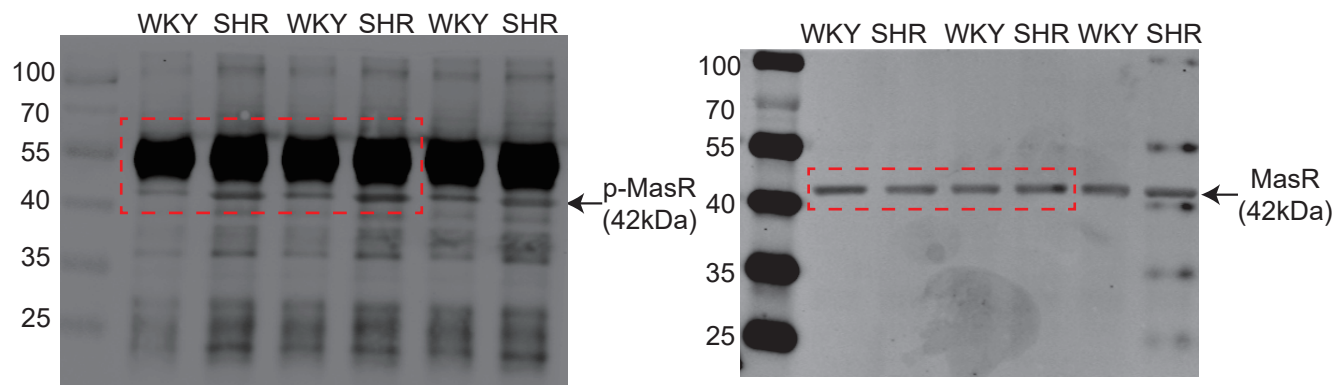

Figure 2C

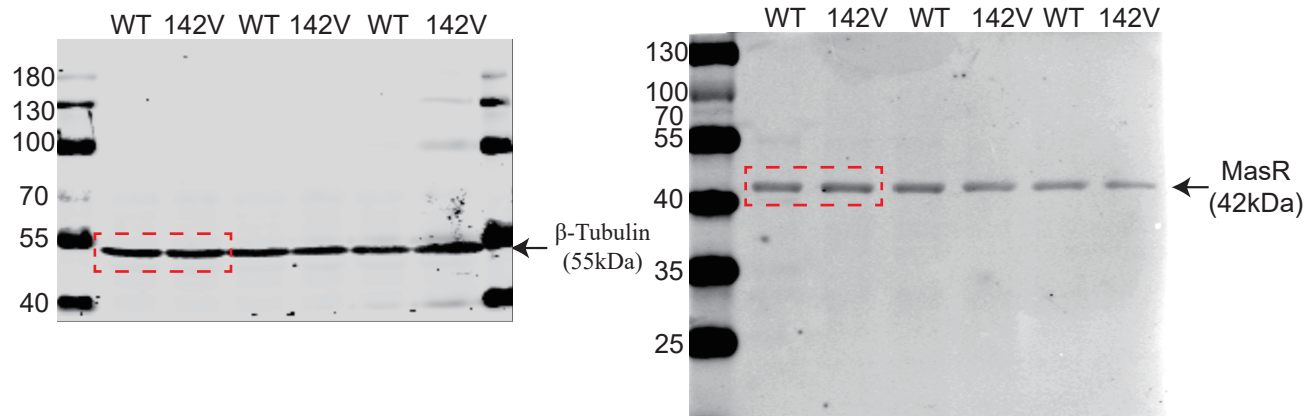

Figure 2D

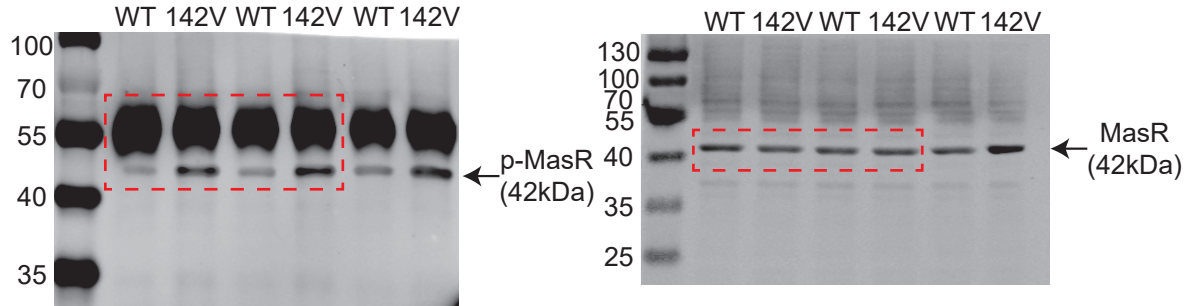

Figure 3C

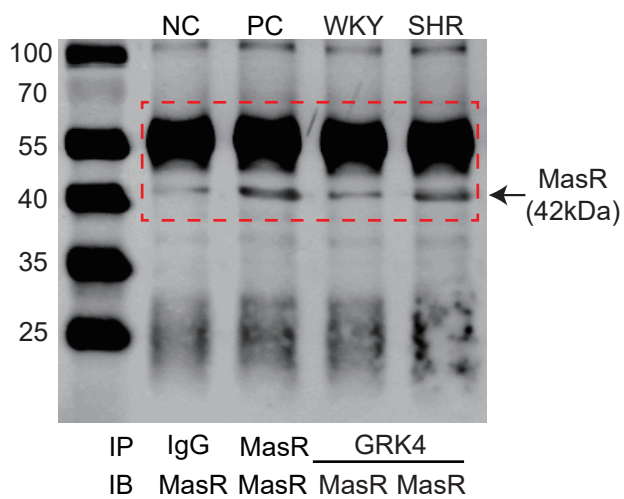

Figure 4C

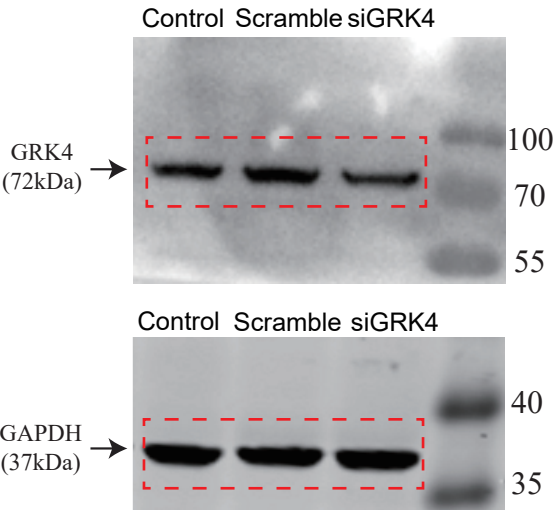

Figure 4D

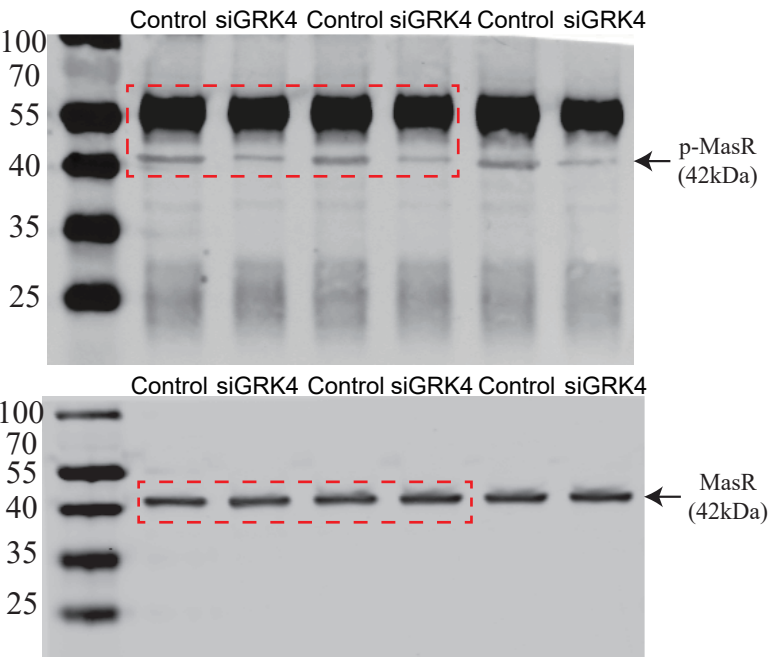

Figure 5B

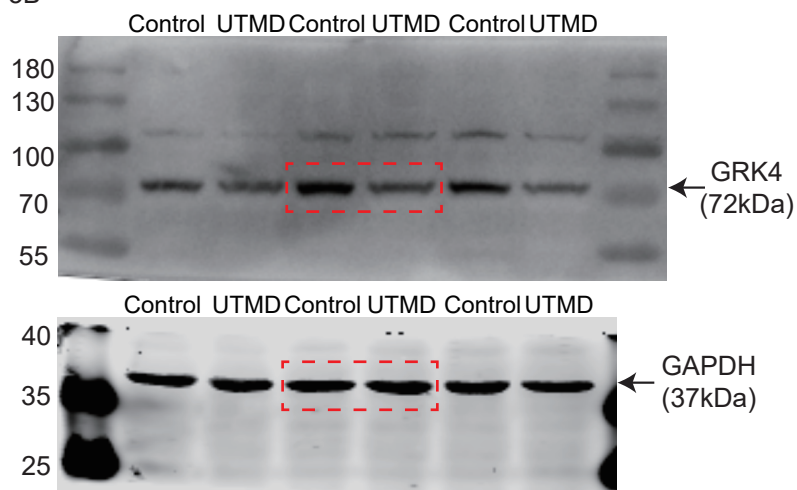

Figure 5C

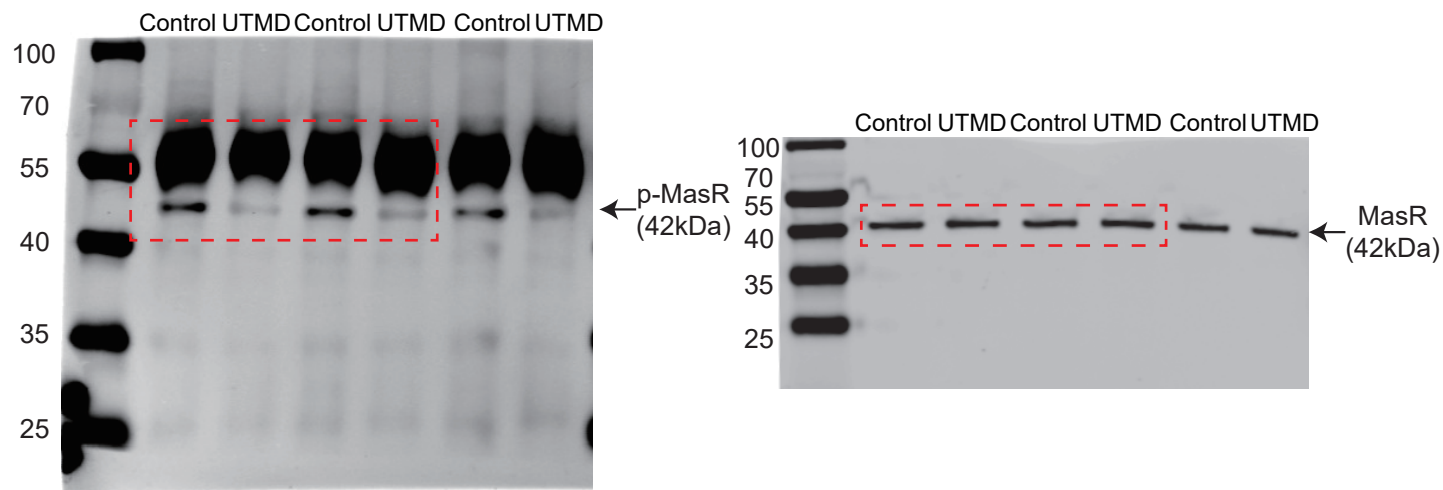

Figure 5E

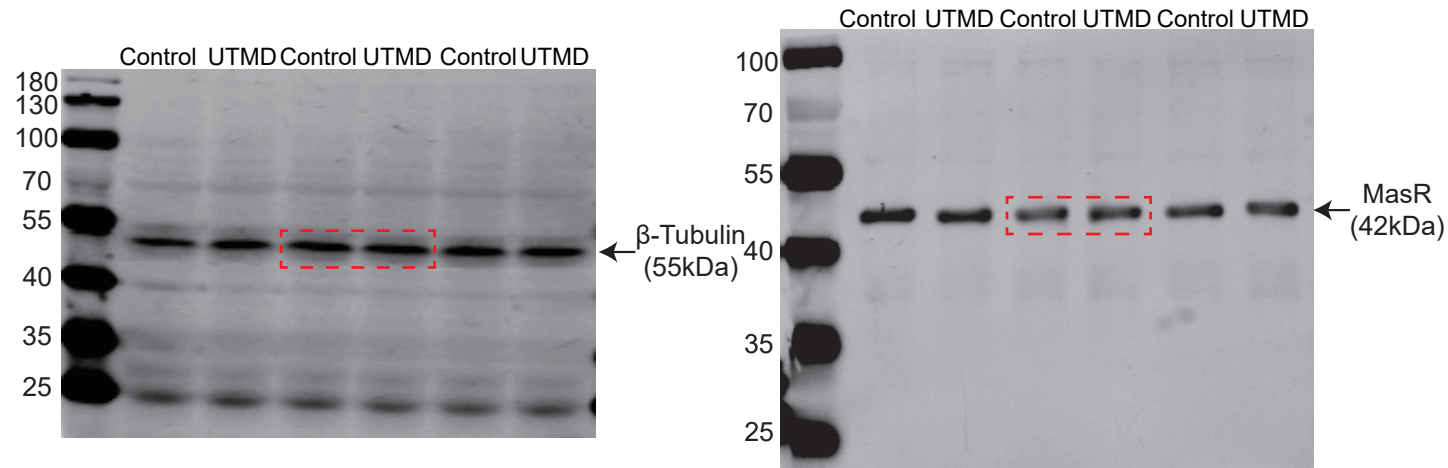

Supplement: S1 Raw Images — (PDF) [file pone.0329547.s004.pdf]
